# A computational cognitive model of behaviors and decisions that modulate pandemic transmission: Expectancy-value, attitudes, self-efficacy, and motivational intensity

**DOI:** 10.3389/fpsyg.2022.981983

**Published:** 2023-01-13

**Authors:** Peter Pirolli, Christian Lebiere, Mark Orr

**Affiliations:** ^1^Institute for Human and Machine Cognition, Pensacola, FL, United States; ^2^School of Computer Science, Carnegie Mellon University, Pittsburgh, PA, United States; ^3^Biocomplexity Institute, University of Virginia, Charlottesville, VA, United States

**Keywords:** cognition, non-pharmaceutical interventions, COVID-19, basic reproduction number, ACT-R

## Abstract

We present a computational cognitive model that incorporates and formalizes aspects of theories of individual-level behavior change and present simulations of COVID-19 behavioral response that modulates transmission rates. This formalization includes addressing the psychological constructs of attitudes, self-efficacy, and motivational intensity. The model yields signature phenomena that appear in the oscillating dynamics of mask wearing and the effective reproduction number, as well as the overall increase of rates of mask-wearing in response to awareness of an ongoing pandemic.

## 1. Introduction

Public health response options to pandemics such as COVID-19 are greatly influenced by predictive epidemiological models (Adam, 2020; Pirolli et al., 2020, 2021; Cramer et al., 2022). Non-pharmaceutical interventions, such as mask wearing, typically involve attempts to modify human behavior to reduce the routes by which a pathogen is transmitted (West et al., 2020; Harvey et al., 2021). Epidemiological models include little to no refined modeling of the psychology of the people who are being affected by the pandemic and who must decide whether and how to comply with public health guidance and mandates. The National Academies of Sciences, Engineering, and Medicine (Brossard et al., 2020) emphasized the importance of psychological science to the mitigation of the spread of COVID-19.

We argue that major advances in computational cognitive modeling are needed to serve as a foundation for predictive and causal-explanatory models of human behavior change in response to pandemics. Such models are needed to shape the course of human behavior response in more precise and less burdensome ways. The COVID-19 pandemic has involved historically the most massive set of natural experiments aimed at changing human behavior along with staggering amounts of data relevant to understanding human behavioral response to the perceptions about the pandemic and related interventions. The availability of these data provides us with an opportunity to understand behavior-change with predictive, explanatory, computational cognitive models. Our preliminary research (Pirolli et al., 2020, 2021) on cognitive models of pandemic behavior response to COVID-19 focused on modeling beliefs, attitudes, intentions, and behavior that are assumed to influence the transmission of COVID-19. Our approach uses Psychologically Valid Agents (PVAs) implemented in the ACT-R (Adaptive Control of Thought-Rational) architecture (Anderson et al., 2004), with input drivers induced from heterogeneous sources including online media such as Twitter that provide indicators of pandemic awareness, beliefs, and attitudes (Pirolli et al., 2020).

In this paper, we present a computational cognitive model that incorporates and formalizes aspects of theories of individual-level behavior change (Fishbein and Ajzen, 1975; Ajzen, 1991, 1998; Brewer and Rimer, 2008; Michie et al., 2013, 2014, 2017; Ajzen and Kruglanski, 2019; West et al., 2020). Such formalization includes addressing the psychological constructs of attitudes (Hunter et al., 1984; Lorenz et al., 2021), self-efficacy (Bandura, 1977; Bandura, 1998), and motivational intensity (Kukla, 1972; Silvestrini et al., 2022). In most cases, these psychological theories and constructs have been verbally specified, although some have been specified with a mathematical foundation (e.g., Hunter et al., 1984; Ajzen, 1991) or in computational agent-based models (e.g., Silvestrini et al., 2022). This paper is devoted to the development of a computational cognitive model that integrates these theories and constructs in way that is predictive of behavior, dynamical (i.e., changing with time and context), and grounded in established cognitive mechanisms. Such models could provide the foundation for understanding and predicting individual-level or aggregate behavior change and could serve as the basis for a variety of population-level modeling techniques, for example, by being embedded in agent-based models (Verelst et al., 2016) or other epidemiological models (Eubank et al., 2004). In addition, their mechanistic underpinning could provide the basis of what-if counterfactual modeling of the effects of various public health actions to non-pharmaceutical interventions and the evaluations of alternative scenarios and strategies.

## 2. Computational modeling approaches to behavior change

### 2.1. The central role of human decision making and behavior in SARS-CoV-2 transmission

In the early months of the COVID-19 pandemic, before the availability of vaccines when NPIs (non-pharmaceutical interventions) where the only available public health action West et al. (2020), pointed out that human behavior is central to the transmission of the SARS-CoV-2 virus. Although our understanding of the transmission routes from one person has become more refined since early 2020, it was understood that behaviors such as social distancing, hand washing, surface cleaning, mask wearing, and reduced touching of the face blocked these transmission routes.

Subsequent research corroborated the impact of social distancing (Gollwitzer et al., 2020) and mask wearing (Howard et al., 2021; Huang et al., 2022) on reducing COVID-19 cases. Because of the central role of behavior in controlling transmission, and the lack of solid empirical evidence for how to promote specific behaviors such as mask-wearing and social distancing, West et al. (2020) advocated for the application of behavior change theory and principles. Similar arguments have been put forth for applying habit formation theory and principles (Harvey et al., 2021).

As vaccines became widely available, the issue of vaccine hesitancy became a major problem for public health officials. Getting vaccinated can be viewed as the result of an individual-level decision-making process (Peretti-Watel et al., 2015) influenced by numerous factors. Empirical research suggests that theories of individual-level behavior change theories explain 43 to 69% of the variance in vaccine hesitancy (Hossain et al., 2021; Wolff, 2021).

### 2.2. Individual behavior change theory

Individual-level health behavior theories (Brewer and Rimer, 2008) include the Transtheoretical Model (Bridle et al., 2005), the Health Belief Model (Harrison et al., 1992), Goal Setting Theory (Locke and Latham, 2002), and the Theory of Planned Behavior (Ajzen, 1991; Ajzen, 1998). Michie et al. (2014) have performed an enormous survey of the behavior change literature and identified 83 theories, 26 mechanisms of action, 93 behavior change techniques, and 1,725 theoretical constructs. A recent meta-analysis (Samdal et al., 2017) of the literature on behavior change techniques identified in Michie et al. (2014) summarizes the evidence on which techniques produce reliable effects along with effect size estimates. As noted above, individual health behavior change theories are typically not formally specified as fine-grained predictive and dynamical models of behavior change.

Rather than provide a survey of these individual-level health behavior theories, we focus on the Theory of Planned Behavior (TPB, 9) as a canonical example of such theories to provide a framework for our discussion of our ACT-R cognitive model. TPB has been studied extensively (Brewer and Rimer, 2008) and meta-analyses support the efficacy of the approach in predicting behavior at a coarse-grained level (Armitage and Conner, 2001). It is implicated as being the most predictive of vaccine hesitancy (Hossain et al., 2021; Wolff, 2021). The TPB proposes that the predictors of a person engaging in a target behavior include the person’s *intention* to do the desired behavior and their *perceived behavioral control*—whether the person perceives themselves as being in control of doing the target behavior. We conceive of intention as specifically a goal intention: The person’s goal to perform a specific behavior. Perceived behavioral control encompasses the concept of *self-efficacy* (Ajzen, 2002), which we discuss in further detail below. In TPB, the predictors of intention are attitudes, subjective norms, and (again) perceived behavioral control. *Attitudes* are whether a person is in favor of doing the behavior. *Subjective norms* are how much the person perceives social pressure to do the behavior. Attitudes, subjective norms, and perceived behavioral control are all forms of *expectancy-value* judgments deriving from beliefs about outcomes, significant referents, and specific facilitating/inhibiting factors, respectively.

Although our ACT-R models have been shaped by TPB, we claim that the ACT-R models themselves incorporate many more additional theoretical mechanisms and assumptions that derive from the ACT-R theory of cognition. There are many criticisms of TPB as a theory (e.g., Sniehotta et al., 2014). TPB is primarily a theory of volitional decision making, but ACT-R is a dual process theory (Kahneman, 2011) that includes unconscious as well as deliberative influences on decisions and behavior. TPB is primarily a static causal influence model, whereas ACT-R is inherently a continuous-time, dynamical model of cognition. The ACT-R models provide a way of capturing the evidenced effects of individual-level experiences and behaviors on future cognitions and future behavior. In sum, TPB has been as useful framework in shaping the ACT-R models presented here, but the models are much more than straightforward instantiations of TPB.

### 2.3. Previous work on cognitive models of COVID-19 behavior change

In previous research, we performed PVA simulations (Pirolli et al., 2020, 2021) to demonstrate the feasibility of mining online media to seed computational models of behavior-change for NPIs (e.g., mask-wearing; social distancing), predicting the timeseries of behavior change for different US regions, and connecting that to epidemiological indicators such as *R_t_* (effective reproduction number). That research developed a framework that integrates multi-level cognitive and social simulation with information networks analysis and epidemiological predictions. The PVAs were initialized and driven using techniques that extract indicators of pandemic awareness, beliefs, and attitudes from online media and available COVID-19 datasets (including polling and epidemiological data). Pirolli et al. (2021) modeled mask-wearing behavior in four states of United States (CA, FL, PA, and NY). Two analyses of COVID-19 Twitter datasets provided inputs to the PVA models. The first Twitter analysis concerned pro- vs. con-mask-wearing analyzed using hashtags. A second Twitter analysis provided a more refined analysis of cognitive content using Natural Language Processing (NLP) techniques. These analyses were input into PVA models of mask-wearing attitudes and behaviors and predictions were compared to mask-wearing behaviors in those four states over the 2020–2021 time frame.

## 3. A computational cognitive model of behavior modulating COVID-19 transmission

### 3.1. Computational cognitive models using the ACT-R theory

ACT-R (Anderson and Lebiere, 1998; Anderson et al., 2004) is a cognitive architecture, i.e., a computational implementation of a unified theory of cognition (Newell, 1990). Unified theories of cognition specify how the structure and dynamics of the brain give rise to the functioning of the mind. Cognitive architectures include mechanisms and representations abstracted from human behavior, arranged as fine-grained interactions between functional modules that reflect the structure and operation of the human brain. A wide variety of cognitive architectures have been proposed over the last five decades since the concept was proposed as a unification of functionality-specific models to provide an integrated account of human cognition (Newell, 1973). Recently, an attempt has been made to extract an emerging consensus regarding the central structures and processes of cognitive architectures in the form of a Common Model of Cognition (CMC), initially called the Standard Model of the Mind (Laird et al., 2017). ACT-R provides a computational implementation of the CMC informed by the rational analysis of cognition (Anderson, 1990) that assumes that our cognitive mechanisms and representations have adapted to the statistical structure of our environment. This assumption enables the development of models based on the cognitive architecture that abstract over details of our personal environment to generate behaviors that respond to the overall regularities of our information landscape.

The ACT-R theory (Anderson, 2007) has evolved since the 1970s to address a wide variety of experimental results on problem solving, decision making, memory, learning, cognitive skill acquisition, perception, and attention, as well as the fine-grained time course of neural processes. The theory has been applied to a variety of domains including computer tutoring systems, human-computer interaction, and language learning. ACT-R is implemented as a simulation environment with a number of software variants of that environment that can simplify application to a specific domain or problem. Practically, ACT-R is a computational cognitive architecture that supports the development of models. A scientific understanding of behavior change in response to pandemics requires such unified models and toolkits. The literature on behavior change is extensive, lacks coherence, and needs mechanistic theory. Preliminary integrative models of behavior change have been developed in ACT-R (Pirolli, 2016a; Pirolli et al., 2018), which provide some promise of their utility to modeling behavior change during a pandemic.

ACT-R is composed of *modules*, processing different kinds of content, which are coordinated through a centralized *procedural module*. Each module corresponds to a brain region. Each module is assumed to access and deposit information into *buffers* associated with the module, and the central *procedural module* can only respond to the contents of the buffers. The procedural module matches the contents of other module buffers and coordinates their activity using *production rules*, which are pairs of conditions and associated actions. Neurally, a production rule is a formal specification of the *flow of information* from buffers in the cortex to the basal ganglia and back again. Productions have a *utility* property that is used to select the single rule that is executed at any point in time.

For the cognitive model presented in this paper, we rely on the *declarative module*, *retrieval buffer*, and *blending buffer* that are used to simulate how people retrieve knowledge and past experiences from long-term declarative memory. We use the ACT-UP simulation system (Reitter and Lebiere, 2010) that implements this specific subset of ACT-R. Knowledge and experience in the declarative module are represented formally in terms of chunks (Miller, 1956; Simon, 1974*)*. Chunks have *activation* levels that determine the probability and time course of chunk retrieval into a buffer. Chunk activations are real-valued quantities produced by *subsymbolic mechanisms* in ACT-R. These subsymbolic mechanisms reflect neural-like processes that determine the time course and probability of cognitive activity and behavioral performance. The dynamics of declarative memory retrieval and production selection are determined by these subsymbolic mechanisms.

Table 1 presents a subset of the ACT-R subsymbolic mechanisms relevant to the current model. The first three equations in Table 1 define how the level of activation of chunks in memory relates to the probability of their retrieval at any given time. The fourth equation defines how activation levels are increased by repeated experiences, or decay with time (forgetting). These first four subsymbolic mechanisms are crucial to the ACT-R model discussed below. A few general comments can be made about these mechanisms. The base-level learning equation and activation equation captures two key memory phenomena: activation increases with the frequency of experience (i.e., a practice effect) and decreases with time (i.e., forgetting). Level of activation dictates retrieval probability and weighs how blended retrievals produce aggregate values over past experiences.

**Table 1:** Core ACT-R mechanisms used in the simulations.

Mechanism	Equation	Description
Blended retrieval	$V=argmin\;\underset{i}{\sum }P_{i}{\left(1-Sim\left(V,V_{i}\right)\right)}^{2}$	***P***_***i***_: Probability of declarative retrieval***Sim (V*,*V***_***i***_): Similarity between compromise value *V* and retrieved value *Vi*
Retrieval probability	$P_{i}=\frac{e^{A_{i}/s}}{\sum_{j}e^{A_{j}/s}}$	***P_i_***: The probability that chunk *i* will be recalled***A_i_***: Activation strength of chunk *i****∑A_j_***: Activation strength of all of eligible chunks *j***s:** Chunk activation noise
Activation	$A_{i}=B_{i}+\varepsilon_{i}$	***B_i_***: Base-level activation reflects the recency and frequency of use of chunk *i****ε***_***i***_: Random noise value
Base level learning	$B_{i}=\ln\left(\sum_{j=1}^{n}t_{j}^{-d}\right)+\beta_{i}$	***n*:** The number of experiences for chunk *i****t***_***j***_: The time since the *j*th presentation***d***: A decay rate***β***_***i***_: A constant offset

### 3.2. Modeling attitudes as the expected value of behaviors

Attitudes are assumed by many (Ajzen, 1991) to be an expectancy-value assessment, such than an attitude *a* towards a behavior is proportional $a\propto \sum b_{i}\;e_{i}$ to the strength of beliefs, *b*, about outcomes and their evaluated values, *e*. For instance, the Theory of Planned Behavior—discussed above—is historically related to seminal work on expectancy-value theory in psychology (Fishbein and Ajzen, 1975). The general idea is that people develop expectations about behavioral outcomes as well as subjective values about those outcomes.

Our model of expectancy-value judgments assumes that decisions and enacted behavior have values that reflect subjective utility—e.g., a degree of satisfaction, reward, or degree of preference (Luce, 1959). That is, when a behavior occurs in some situation and produces an outcome, it may be associated with a subjective assessment of its value. Based on ACT-R, we assume those experienced associations of features as <situation context, behavior, outcome, value> associations that are stored in declarative memory as chunks. These instances of decision and behavioral experience are core to the theory of Instance Based Learning that has been developed within ACT-R (Gonzalez et al., 2003). Over time, implicit knowledge about decision making is generated through the creation and storage of experiential instances.

Decision-makers retrieve and generalize from these instances to evaluate alternatives, make a decision, and execute a behavior. This is achieved through memory retrieval and blending. Memory retrieval in ACT-R is a request to retrieve a specific memory chunk when provided with a set of cues (features). For example, a set of features might in a situational context might be used to retrieve a memory of a behavior that occurred in a similar situation. A blended retrieval produces a memory that aggregates and generalizes over past experience based on inter-instance similarity and the activation of those memories. For instance, a blended retrieval of the subjective value of a behavioral outcome is an aggregate of instance values, weighted by the activation of those instances (see Table 1).

**Figure 1:**
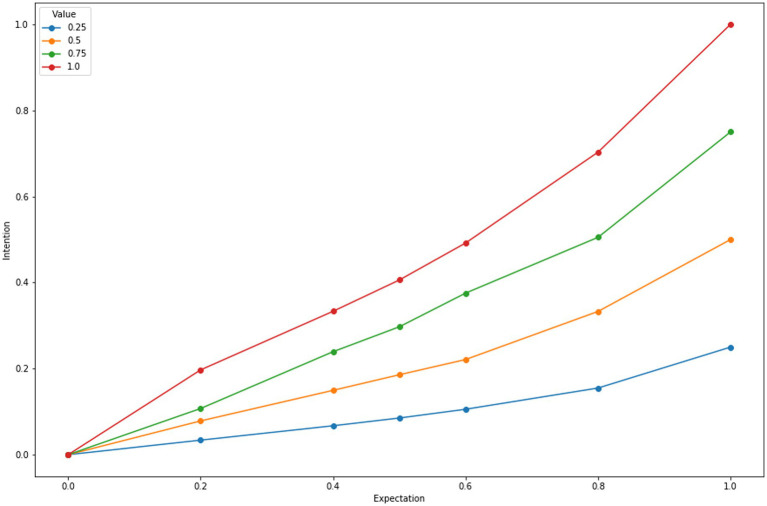
Expectancy value assessments for multiple simulation runs of ACT-R in which subjective values associated with success range from 0.25 to 1.0, and the probability of success ranges from *p* = 0 to 1.0.

Figure 1 illustrates how instance-based learning mechanisms yield expectancy-value judgments. The data in Figure 1 come from a purely synthetic set of simulation runs. For each run, there is a training phase in which experience instances of <situation, behavior, value> are stored in memory, followed by a test in which a blended retrieval is made to judge the expected value of a behavior. The behavior is arbitrarily labelled as “mask wearing.” Each training run simulates a behavior producing a value = *v* with probability = *p* or a value = *0* with probability (1-*p*) for a total of 100 experiences (instances). Note that there is no explicit representation of probability—just a set of experiences that produce subjective values with some probability. Following, a training run, a blended retrieval is performed to assess the expected value of “mask wearing.” The synthetic runs in Figure 1 range over *p* = 0, 0.2, …1.0 and *v* = 0.25, 0.5, …1.0.

### 3.3. Modeling the time and frequency effects of messaging or experiences

**Figure 2:**
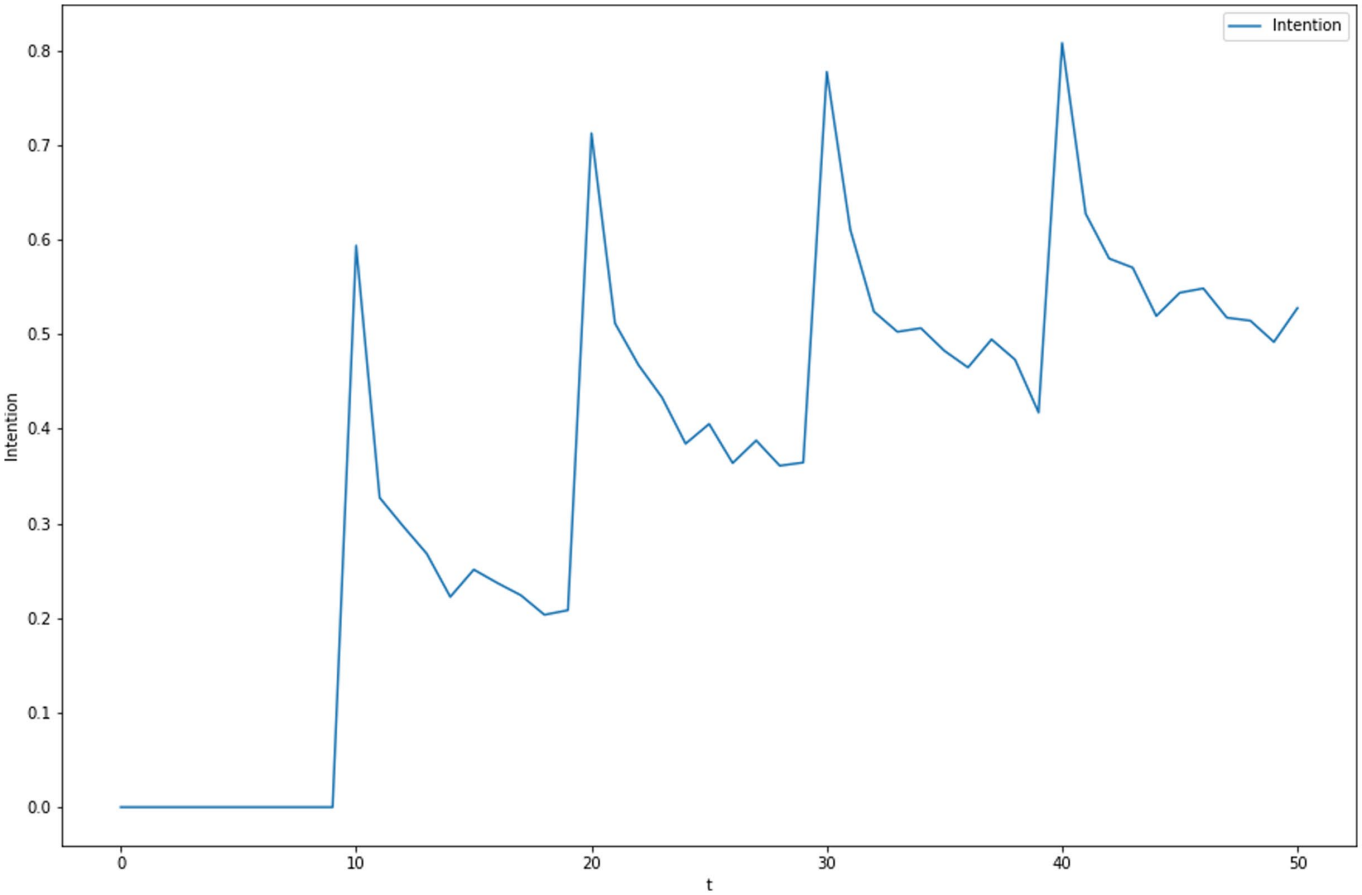
Dynamics of subjective intentions that start a level of zero but messaging at *t* = 10, 20, 30, 40 promotes a higher level of intention.

**Figure 3:**
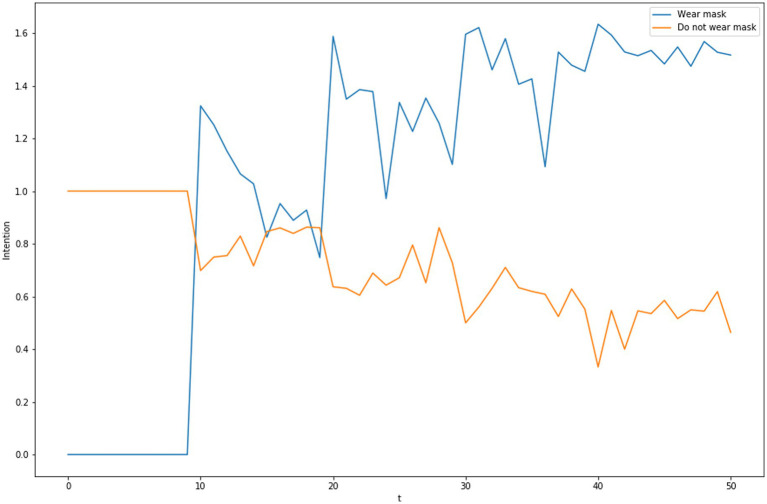
Dynamics of subjective intentions for mask wearing or not wearing masks with messaging promoting masks at *t* = 10, 20, 30, 40.

**Figure 4:**
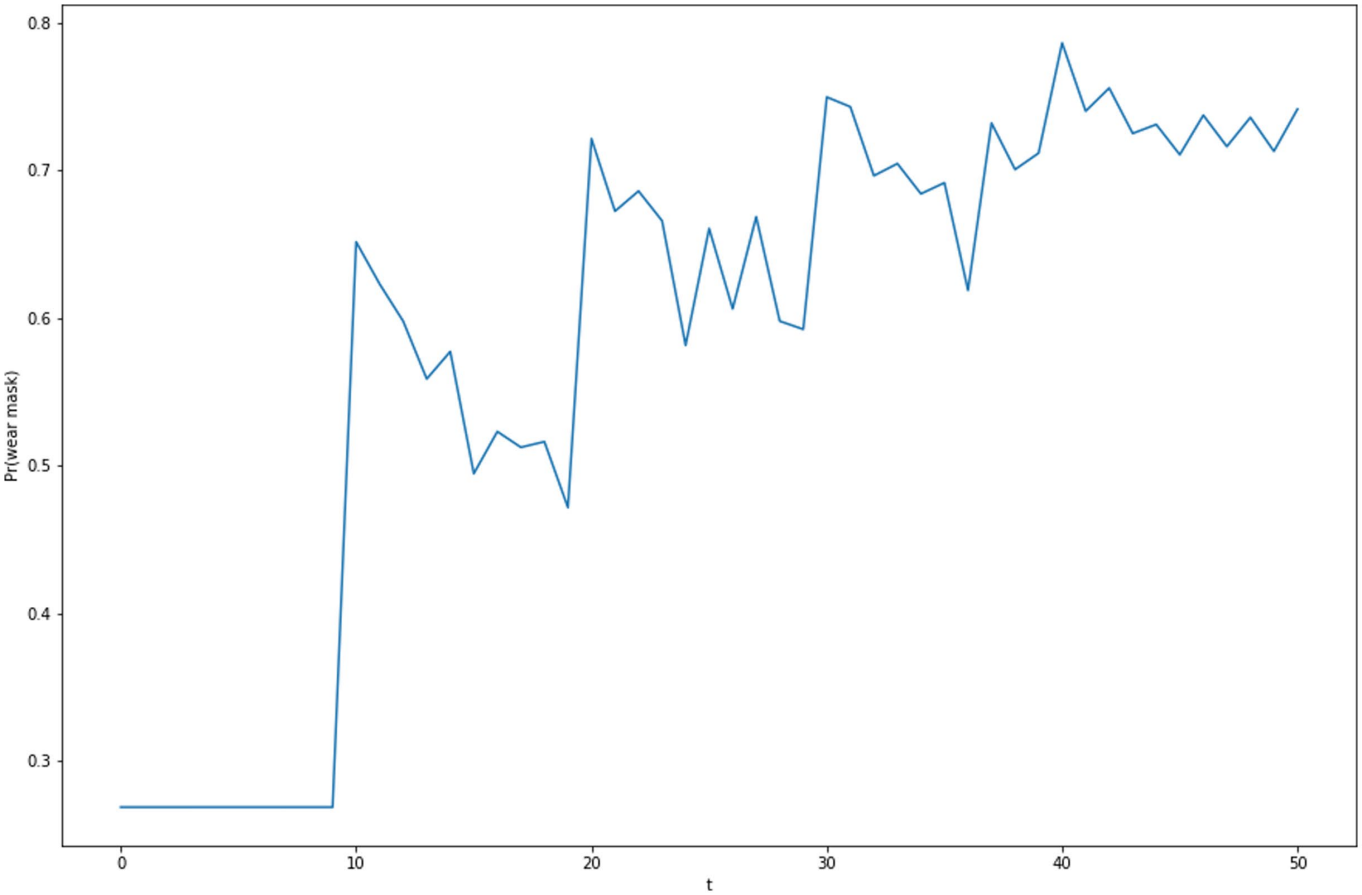
The impact of the changes of intentions in Figure 3 on mask choice probability.

Attitudes can be modified through messages and experiences, and the impact of messages can be modulated by evaluations of the credibility of the sources of those messages (Hunter et al., 1984). Figures 2–4 illustrate the predicted dynamics of message effects on intentions in another series of ACT-R simulations. The simulations assume that the subject value of “mask wearing” is initially zero, and messages are delivered at specific points in time that ‘mask wearing’ has a higher value. Those messages are stored as additional instances in memory and affect subsequent judgments based on blended retrievals. In Figure 2, one can observe the effects of the base-level learning mechanism: (Adam, 2020) As the cumulative frequency of messages increases, the activation of chunks representing a higher value for “mask wearing” increases, and that weights the expected value assessments to be larger (Pirolli et al., 2020), the effect of a message decays with time, again because of the decay in activation specified by base-level learning.

So far, we have simplified the discussion by only attending to one behavioral alternative (“mask wearing”), but decision making involves choice amongst multiple alternatives. Figure 3 presents data from simulation runs in with there are alternative behaviors (“wear mask,” “do not wear a mask”) in which the subjective value of “wear mask” is initially less than the alternative, but messages at specific points in time place a higher value on “wear mask” than “do not wear a mask.”

These competing behavior intentions are related to a decision to pursue a behavior using a variation of a Random Utility Model (McFadden, 1974) or Luce’s Choice Axiom (Luce, 1959) by which the probability of choosing an alternative with an intention of *i* from a set of alternatives having intentions *J* is given by:

$$Pr\left(i|J\right)=\frac{\exp\left(i\right)}{\sum_{j\in J}\exp\left(j\right)}$$

Using this choice probability model, the competing intentions in Figure 3 produce the choice to “wear mask” with the probabilities depicted in Figure 4.

### 3.4. Modeling self-efficacy and motivational intensity

Self-efficacy defined as an individual’s belief in their capacity to execute behaviors necessary to produce specific performance attainments. The Social Cognitive Theory of self-efficacy (Bandura, 1998) predicts that behavioral goals that are perceived as too difficult are unlikely to be attempted. In general, greater levels of self-efficacy lead to greater likelihoods of achieving a goal. Self-efficacy is often broken down into: (Adam, 2020) perceived general self-efficacy, which is an individual’s perception of their ability across many situations and (Pirolli et al., 2020) task-specific self-efficacy, which is an individual’s perception of their ability to perform a specific action or task in one or a variety of situations. ACT-R models of self-efficacy have focused on the task-specific self-efficacy. However, because of the way declarative memory blending works in ACT-R these models do have the capability to transfer self-efficacy across similar behaviors and tasks or produce self-efficacy across a range of situation bs and or tasks. ACT-R models (Pirolli, 2016a,b) are based on self-efficacy from mastery experiences (i.e., personal experiences of success), but in principle can be based on vicarious experience (e.g., witnessing similar others who have exhibited mastery).

Related to assessments of self-efficacy is how motivated people are to engage in effortful behaviors (physical and or cognitive). As Vancouver (Vancouver, 2008; Vancouver et al., 2008) has noted, when self-efficacy is low relative to a difficult task, then it is likely to be judged as being more effortful, so high motivated effort can be compensatory for low self-efficacy—up to a limit. This is because the general observation and Motivational Intensity Theory (Silvestrini et al., 2022) is that as the difficulty of a task or behavior exceeds some threshold, people will not be motivated to allocate effort, producing a saw-tooth relationship between effort and perceived task difficulty. Previous ACT-R research (Pirolli, 2016b) combined self-efficacy theory with an implementation of the Attributional Theory of Performance (Kukla, 1972), which is a variant of Motivational Intensity Theory.

How ACT-R integrates self-efficacy and motivation intensity theories can be illustrated with another synthetic example about mask wearing. The ACT-R theory (Pirolli, 2016b) assumes that when mask wearing is judged as having a difficulty, δ, a self-efficacy *θ(t*) at time *t*, and the individual engages with motivation intensity τ(*t*), then the probability of engaging in the behavior will be another variation of Luce’s Choice Axiom:


$$Pr\left(engageinbehavior\right)=\frac{\exp\left(\delta -\theta \left(t\right)+\tau \left(t\right)\right)}{1+\exp\left(\delta -\theta \left(t\right)+\tau \left(t\right)\right)}$$

As described by Pirolli (2016a) this ACT-R model assumes that self-efficacy and intended effort are fundamentally the result of memory processes. Past experiences of efficacy at behaviors similar to a target goal are retrieved and blended together to produce assessments of self-efficacy and intended effort for the new goal. The assumption is that given a decision to pursue a goal, an assessment is made of the difficulty, δ, of achieving that goal. A blended retrieval is performed to assess the self-efficacy, *θ(t*), based on memory of experiences on behaviors similar to the goal behavior. A judgment is made about the intentional level of effort required to achieve a behavior with desired probability *p*:


$$\tau \left(t\right)=\ln\left(\frac{p}{1-p}\right)\delta -\theta \left(t\right)$$

It is assumed that the individual will put in effort τ(t) if it is less than a threshold ϕ.

**Figure 5:**
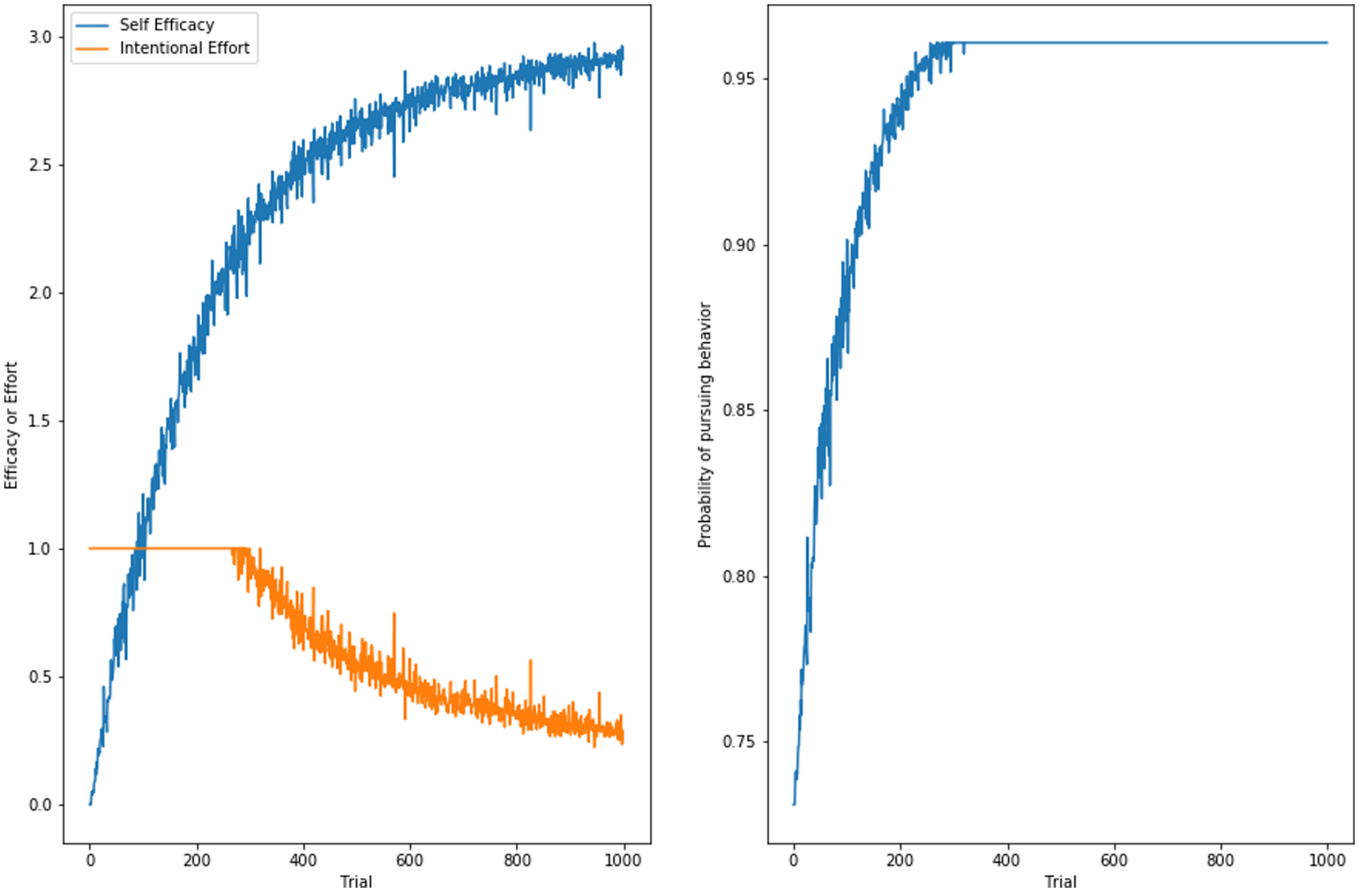
The dynamics of self-efficacy and intentional effort over time (left) and the impact on the probability of pursuing a behavior (right).

If the behavior is performed, then a new instance is learned. That instance will be stored with a self-efficacy that includes the old self-efficacy value plus the additional intentional effort expended. New successful experiences on behaviors where the perceived difficulty was high relative to self-efficacy—but within the limits of what a person was motivated to put in the effort required—will tend to improve self-efficacy with repeated experience. Figure 5 depicts another synthetic simulation illustrating the dynamics of self-efficacy, intentional effort, and probability of performing the behavior. Figure 5 show the growth of self-efficacy and diminishment of intentional effort with successful experiences. Figure 5 shows how the probability of engaging with the behavior increases with self-efficacy.

### 3.5. Modeling norms affecting polarization

**Figure 6:**
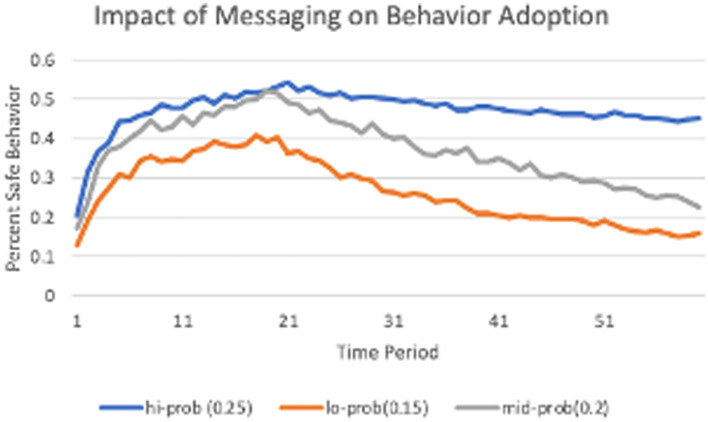
The evolving polarization of behavior adoption in response to messaging.

Compliance with NPIs (and vaccination decisions) involves decision making between safe and risky options. As has been shown empirically by Gollwitzer et al. (2020), the probability of engaging with an NPI behavior can differ initially between groups having different normative beliefs (as measured by political leaning), and those differences can be amplified over time. Figure 6 illustrates this amplification process. The chunks in this model reflect the relative payoff values of the two options of wearing or not wearing mask, including both outcomes of the riskier option (basically usual, unencumbered life vs. illness and potentially death) as well as the single outcome of the safe option (the annoyance of wearing a mask). The relative activations of those options reflect the initial messaging propagated in social and mass media, gradually supplemented by personal experience. A key characteristic of this process of decision-making under risk is that later decisions are influenced by the sampling of options early in the process, itself driven by the initial presentation of the options (Lebiere et al., 2007; Erev et al., 2010). An important consequence is to amplify initial differences in messaging as found in Gollwitzer et al. (2020). The blue line represents counties with a higher proportion of messages (0.25) emphasizing the negative outcome of the risky option. This leads to an expectation of the risky option that is worse than that of the safer option, leading to that option being selected consistently, and the relative expectations being maintained, even when the original messaging is relaxed (period 20). The red line represents counties with a lower proportion of messages (0.15) warning about the negative outcome. This leads to an expectation of the risky option that is often better than the safer option, resulting in that risky option being selected with higher frequency. Because the probability of the negative outcome was relatively low, this led to a gradual improvement in expectation leading to lower adoption of the safer option. Once messaging is relaxed, the usual risky behavior then quickly returns as the default option.

### 3.6. Modeling awareness-driven oscillations in behavior

**Figure 7:**
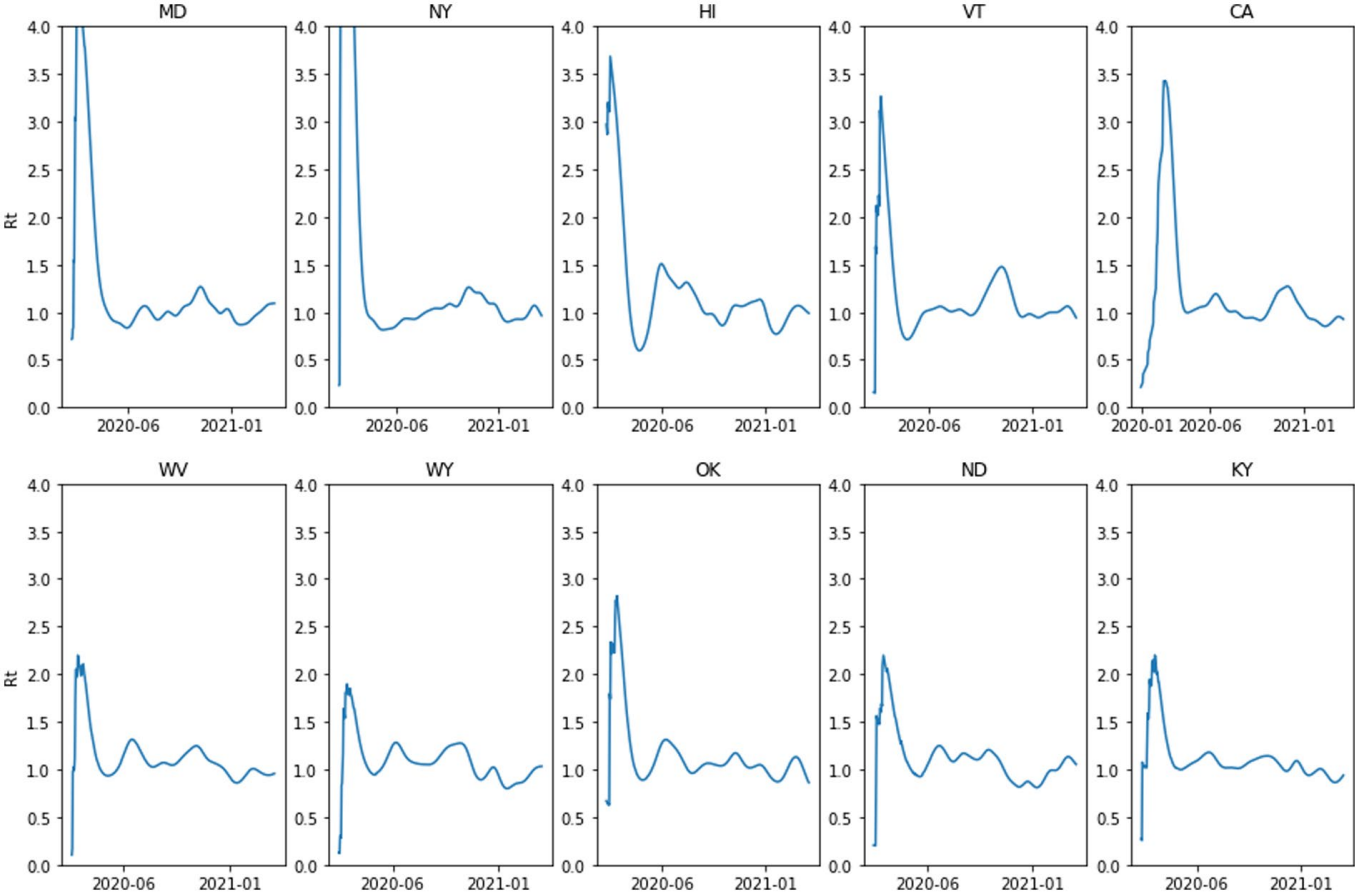
The effective reproduction number for the first three waves of COVID-19 in the United States for the top five states that voted for the 2016 Republican presidential candidate (bottom) and top five that voted for other candidates (top).

It has generally been observed throughout the history of pandemics that people typically modulate their behavior in ways that mitigate transmission rates before NPI mandates (Christakis, 2020). This awareness-driven behavior modulates the shape of epidemiological curves such as case rates or effective transmission rates, *R_t_* (Weitz et al., 2020). It has been argued (Weitz et al., 2020) that it is awareness-driven behavior that produces the signature temporal phenomenon observed in virtually all regions: the damped oscillation pattern of the effective transmission rate, *R_t_*, such as those presented in Figure 7. That is, there is typically a rapid decline from *R_t_* > 1 as people react to the initial spread of the virus, followed by an oscillation around *R_t_* = 1. This oscillation phenomenon is reminiscent of a Proportional-Integral-Derivative control system in which a controlling intervention (e.g., mask-wearing) occurs in proportional response to the state of the system (e.g., *R_t_*), although there may be lags between awareness of the system state and the response, and between the response and effecting a change.

**Figure 8:**
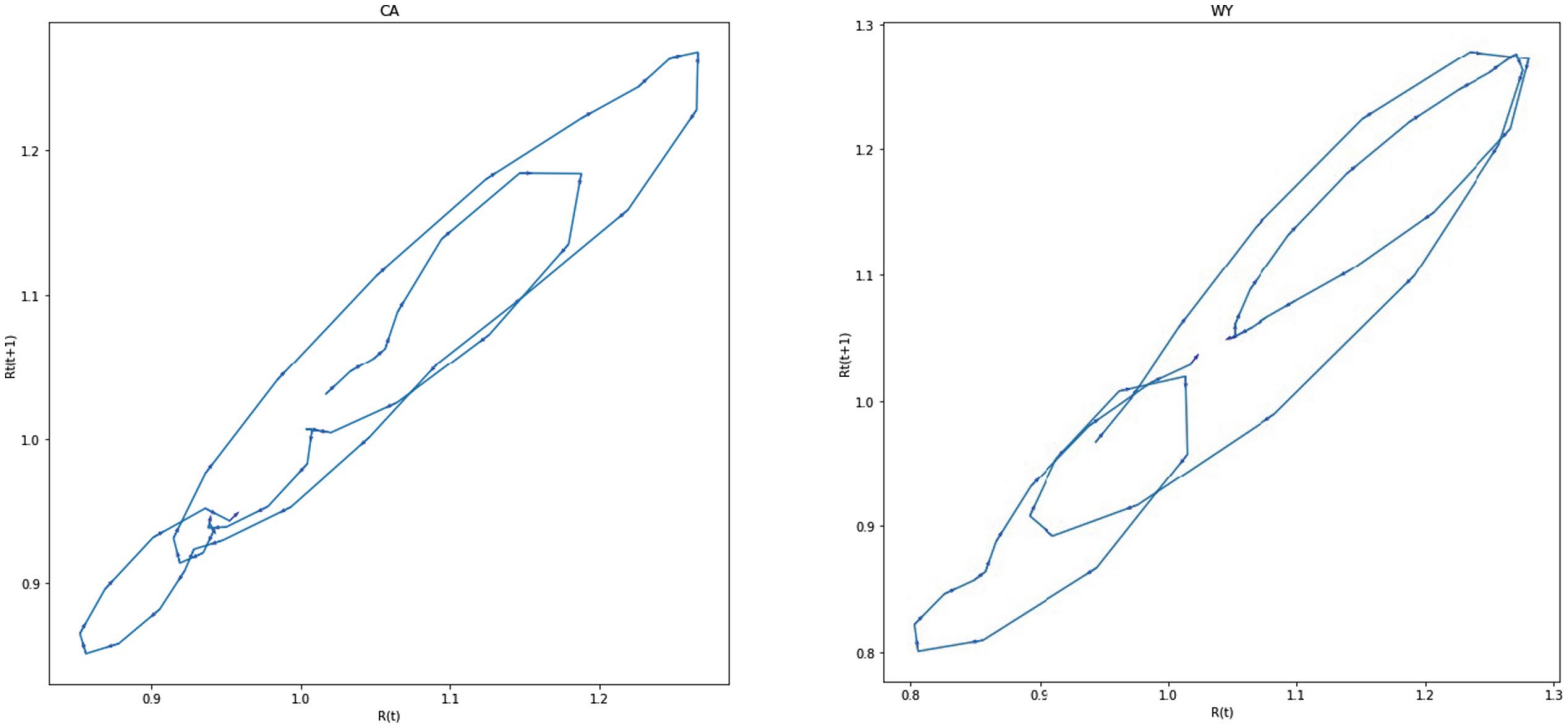
The oscillation of the effective reproduction number in two United States states.

However, the observed oscillation pattern is not entirely simple. Figure 8 presents phase space plots for *R_t_* (*t* + 1) by *R_t_* (*t*) over the first three waves of COVID-19 in the United States for CA and WY (see the Supplementary material for description of the data sources and phase space diagrams for five states with the highest 2016 Republican Presidential vote and five states with the highest 2016 Democrat Presidential vote). In general, one can observe that there is oscillating pattern around the *R_t_* = 1 mark, but in both cases the oscillation loops appear to shift over time.

**Figure 9:**
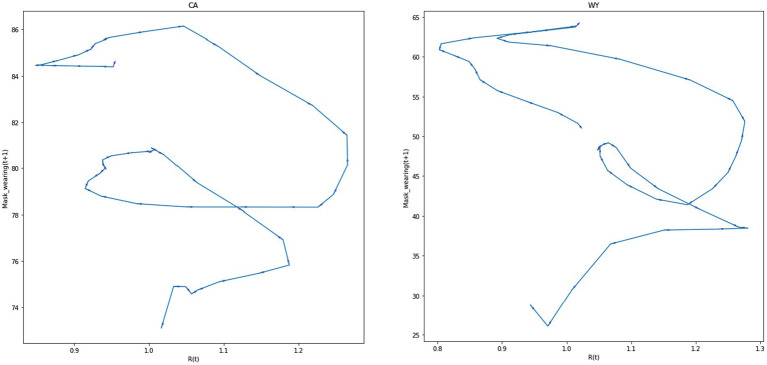
The oscillation of mask wearing and effective reproduction number in two United States states.

Figure 9 plots the phase space relationship between *Rt* and mask-wearing for CA and WY for the first three waves of COVID-19. As can be seen in Figure 9, the mask-wearing response in WY is initially far lower than CA, and the decline in mask-wearing as *R_t_* declines is sharper in WY than in CA, where mask-wearing essentially stays flat as *R_t_* values decrease. We hypothesize that this is the result of to the norm-driven polarization discussed above.

**Figure 10:**
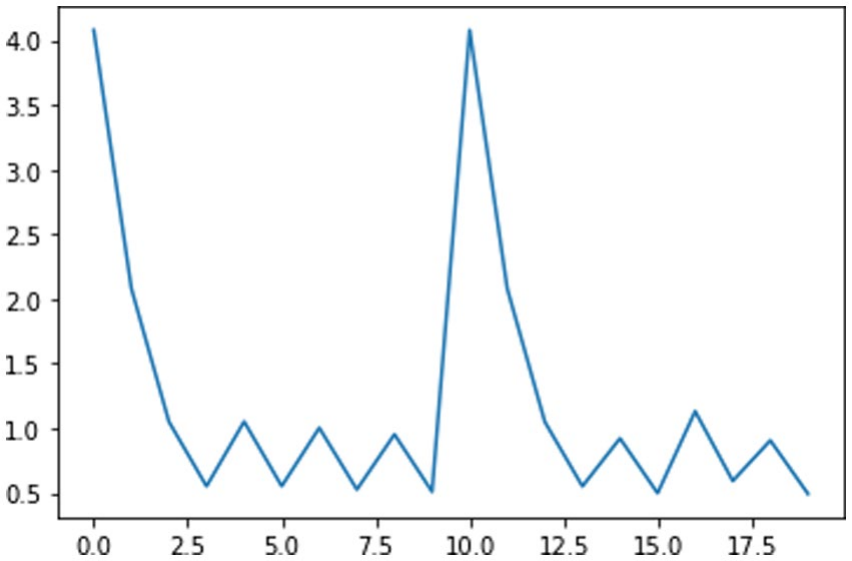
ACT-R simulation of the oscillation of reproduction number over two simulated waves of COVID-19 variants.

**Figure 11:**
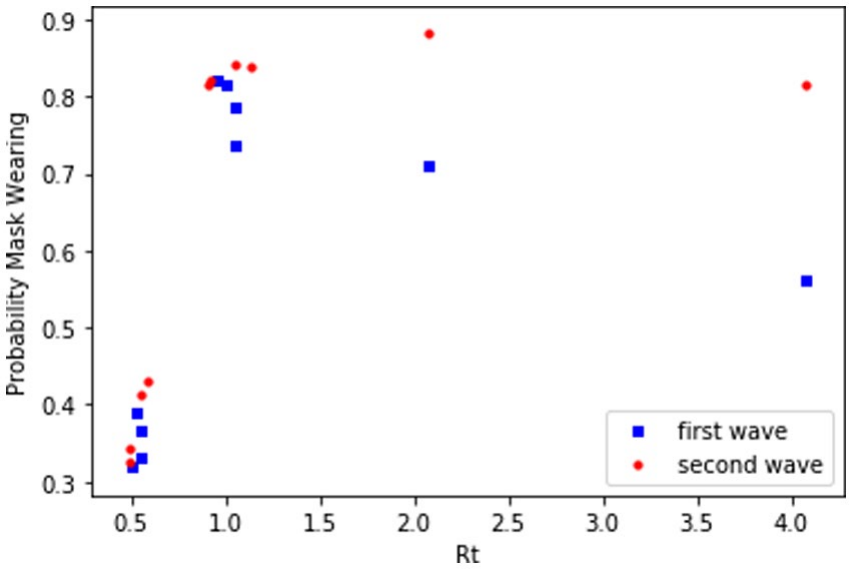
ACT-R simulation produces an overall increase in mask wearing as a function of Rt over two simulated waves of variants.

Figure 10 presents a model of this oscillation pattern that emerges from an integration of expectancy-values attitudes, self-efficacy, intentional effort and the assumption that individual assigns value to mask wearing or not, based on their perceptions of whether the pandemic is increasing or abating. Again, this is a synthetic simulation of two hypothetical waves driven by variants with *R_0_* = 4 and *R_0_* = 5, corresponding to the values estimated for the first wave and delta-variant waves of COVID-19 (Liu and Rocklov, 2021; Yu et al., 2021). High *R_t_* values trigger strong behavior activation, leading to mask wearing, which leads to a decrease in infection. This in turn leads to a relaxation in behavior, triggering another increase and another cycle starts. One effect of the experience built up during the first wave of the simulation is that mask wearing response is more probable during the second wave (Figure 11). This is primarily the result of the build-up of self-efficacy (Figure 11).

**Figure 12:**
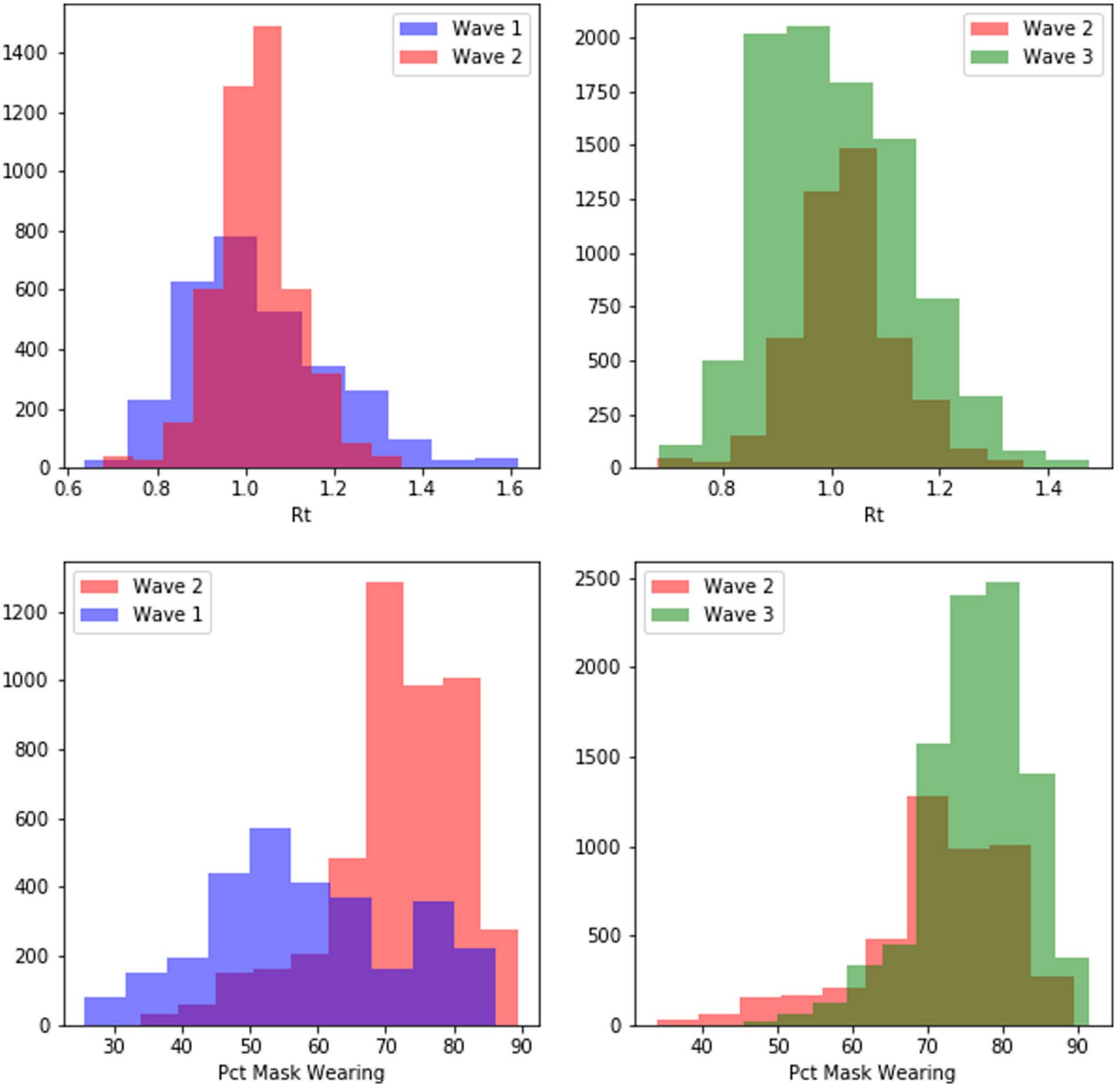
Distribution of effective transmission number over three waves of COVID-19 in the United States (top) and the shift of mask-wearing rates over the same three waves (bottom).

**Figure 13:**
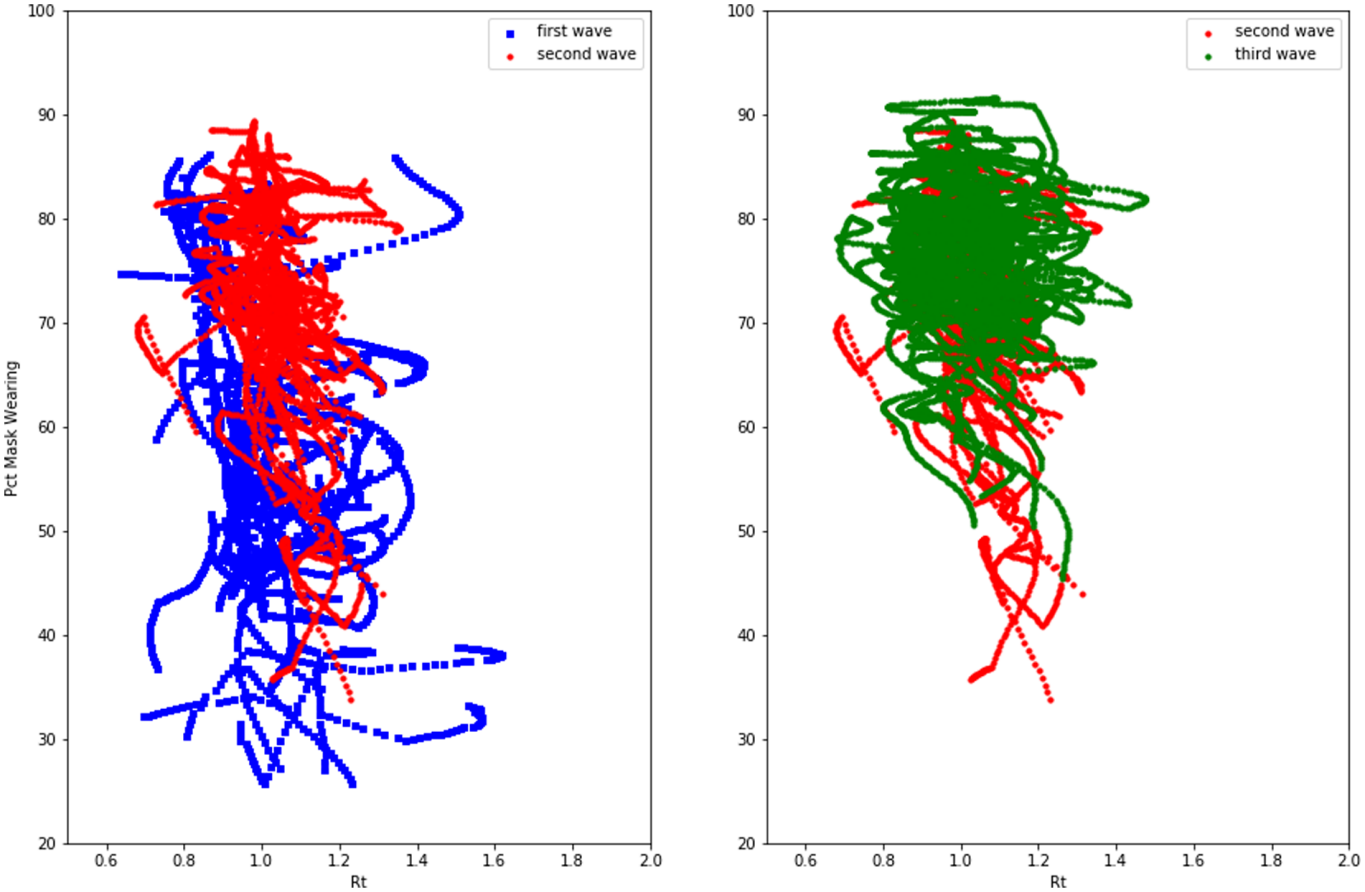
Scatter plot of mask wearing rates by Rt over three waves of COVD-19 in the United States.

So, the cognitive model predicts an increase in mask-wearing probability to perceived COVID-19 transmission rates. That is, for a given value of Rt, the proportion of people who choose to wear a mask should increase with successive waves. Figure 12 shows that the distribution of daily mask wearing per state increased from the first to third waves of the COVD-19 pandemic (first wave mean mask wearing = 58.22%, second wave mean = 71.62%, third wave = 76.46%). More specifically the percentage of people in a state on a given day at a given *R_t_* on that day increased from the first wave to the third wave, as shown in the scatterplots in Figure 13.

## 4. Discussion

In public health practice, behavioral prevention and intervention efforts have relied on the notion that identifying risk-factors of individuals can provide a basis for whom to target, given a specific disease or outcome. This includes social factors (e.g., the position of an individual in a network) as well as individual-based factors (e.g., gender, race/ethnicity, and age). This perspective applies to both acute events (pandemic, environmental emergency) and chronic public health issues (smoking, obesity, etc.). For the latter there is an additional need. Mechanistic models that forecast key outcomes of interest (e.g., force of infection, how many people will accept vaccination) or provide what-if mitigation scenarios (what if we mandate masks) for use by policy makers sometimes require assumptions about behavior change or behavioral compliance (Halloran et al., 2008). The use of social psychological theory is part-and-parcel of practice in public health, yet as it is used today we see several practical limitations: (i) little incorporation of dynamics of behavior, (ii) no affordance for testing an interventions efficacy on behavior change *in silico* (simulation), (iii) lack of a general and unifying model for application across behavioral domains and public health contexts, (iv) little feasibility for incorporation of the theory into mechanistic, real-time forecasting or what-if population models.

Computational cognitive modeling offers one approach to mitigate the limitations of current public health practice. Its foundational advantage is that it represents the detailed mental/cognitive processes and representations that drive behavior. For example, one of our efforts presented above was a model of intention formation that was derived from a detailed specification of the human memory system. The latter was derived from the ACT-R cognitive architecture, a formal theory of the human mind that rests on decades of both experimental and neurophysiological human data. In a sense, our intention model is constrained in its structure and parameterization by decades of abduction between experimental data and psychological theory. This, we argue, is the foundation for the computational cognitive modeling approach in public health.

The computational cognitive modeling approach is extremely well suited to address each of the limitations listed above. First, it affords a clear understanding of the dynamics of behavior change precisely because the basis for ACT-R is theory about the dynamics of human memory, in general. Second, it provides a test-bed for the effects of intervention/prevention efforts (e.g., messaging, or temporal effects of mitigations) *in silico*. ACT-R is a computational theory, and, is naturally extendable to small-scale social simulations of groups for testing in social contexts (Morgan et al., 2021). Third, it is a general approach, unified by the ACT-R cognitive architecture (or even the Common Model of Cognition), that can span behavioral domains (e.g., chronic diseases such as cancer and obesity and infectious disease such as HIV or COVID-19) and span social contexts and differences in built environments). Finally, it can form the basis for the behavior models for large or at-scale simulations of infectious disease and mitigation. Agent-based modeling has been integrated with ACT-R in several contexts (Bhattacharya et al., 2019; Orr, 2019; Orr et al., 2021).

A final point we’d like to make is more general but important for future integration with the public health community. Computational cognitive modes provide a perspective on risk-factors (Orr and Plaut, 2014). Different risk groups have the same mental apparatus; the difference between groups amounts to what has been learned *via* social context and what are the affordances of the built-environment. Cognitive modeling forces one to think through what are the informational and contextual differences between groups and how this has effected the operation of the cognitive model. In short, from the cognitive modeling perspective, all people and groups are the same. Differences in risk behaviors stem from differences in experience. Although our focus here has been on behavioral responses, such as mask wearing, the underling processes are expected to generalize to other important pandemic-related health decisions, especially that of vaccination. This will be a challenge because of the causal role played by individual experiences, beliefs, and values in vaccination decisions (Reimer et al., 2022).

## Data availability statement

The original contributions presented in the study are included in the article/Supplementary material, further inquiries can be directed to the corresponding author.

## Author contributions

PP and CL implemented the ACT-R cognitive model. PP, CL, and MO developed the theoretical mapping from pandemic awareness to behavioral response and contributed to the writing. All authors contributed to the article and approved the submitted version.

## Funding

Portions of the work on this material were supported by the National Science Foundation Grants Nos. 2033390 and 2200112 and the Office of the Director of National Intelligence (ODNI), Intelligence Advanced Research Projects Activity (IARPA), via 2020-20092500001. The views and conclusions contained herein are those of the authors and should not be interpreted as necessarily representing the official policies or endorsements, either expressed or implied, of the ODNI, IARPA, or the United States Government.

## Conflict of interest

The authors declare that the research was conducted in the absence of any commercial or financial relationships that could be construed as a potential conflict of interest.

## Publisher’s note

All claims expressed in this article are solely those of the authors and do not necessarily represent those of their affiliated organizations, or those of the publisher, the editors and the reviewers. Any product that may be evaluated in this article, or claim that may be made by its manufacturer, is not guaranteed or endorsed by the publisher.

## Supplementary material

The Supplementary material for this article can be found online at: https://www.frontiersin.org/articles/10.3389/fpsyg.2022.981983/full#supplementary-material
