# Supplementary material for: A computational cognitive model of behaviors and decisions that modulate pandemic transmission: Expectancy-value, attitudes, self-efficacy, and motivational intensity
Source: Front Psychol. 2023 Jan 13;13:981983. doi: 10.3389/fpsyg.2022.981983 (PMC9880284; doi:10.3389/fpsyg.2022.981983)
Supplement: Supplementary file 1 [file Data_Sheet_1.docx]

Supplementary Material

# Supplementary Data and Code

Analysis and figures are based on data from multiple public sources. Our analysis concerns the time period of the first three waves of Covid-19 as defined by Pew Foundation <https://www.pewresearch.org/politics/2022/03/03/the-changing-political-geography-of-covid-19-over-the-last-two-years/>. A Jupyter python notebook and partly filtered comma-separated files containing the data used in the production of the figures are available from the corresponding author. We are grateful to Anton Gollwitzer who provided access to data presented in Gollwitzer et al. (2020). That dataset provided the county-level and state-level information regarding partisanship (political leaning). *R_t_* data at the state and county level were downloaded from https://covidstim.org , a project of the Yale School of Public Health. Mask-wearing data at the state level was downloaded from the CovidStates project at <https://covidstates.org> (Lazer et al., 2021).

**References**

Gollwitzer, A., Martel, C., Brady, W. J., Pärnamets, P., Freedman, I. G., Knowles, E. D., & Van Bavel, J. J. (2020). Partisan differences in physical distancing are linked to health outcomes during the COVID-19 pandemic. *Nature Human Behaviour, 4*(11), 1186-1197. doi:10.1038/s41562-020-00977-7

Lazer, D., Santillana, M., Perlis, R. H., Quintana, A., Ognyanova, K., Green, J., . . . Gitomer, A. (2021). *The COVID States Project #26: Trajectory of COVID-19-related behaviors*. Retrieved from
